# Delayed onset of tricuspid valve flow in repaired tetralogy of Fallot: an additional mechanism of diastolic dysfunction and interventricular dyssynchrony

**DOI:** 10.1186/1532-429X-13-43

**Published:** 2011-08-24

**Authors:** Ai-Min Sun, Fahad AlHabshan, Michael Cheung, Gabriele Bronzetti, Andrew N Redington, Lee N Benson, Christopher Macgowan, Shi-Joon Yoo

**Affiliations:** 1 Department of Diagnostic Imaging, Hospital for Sick Children, 555 University Avenue, Toronto, Ontario, M5G1X8, Canada; 2 Department of Paediatrics, Division of Cardiology, The University of Toronto School of Medicine, The Hospital for Sick Children, 555 University Avenue, Toronto, Ontario, M5G1X8, Canada

**Keywords:** tetralogy of Fallot, delayed onset of tricuspid valve flow, phase-contrast magnetic resonance, right ventricular function, diastolic dysfunction

## Abstract

**Background:**

Diastolic dysfunction of the right ventricle (RV) is common after repair of tetralogy of Fallot. While restrictive physiology in late diastole has been well known, dysfunction in early diastole has not been described. The present study sought to assess the prevalence and mechanism of early diastolic dysfunction of the RV defined as delayed onset of the tricuspid valve (TV) flow after TOF repair.

**Methods:**

The study population consisted of 31 children with repaired TOF (mean age ± SD, 12.3 ± 4.1 years) who underwent postoperative cardiovascular magnetic resonance (CMR). The CMR protocol included simultaneous phase-contrast velocity mapping of the atrioventricular valves, which enabled direct comparison of the timing and patterns of tricuspid (TV) and mitral (MV) valve flow. The TV flow was defined to have delayed onset when its onset was > 20 ms later than the onset of the MV flow. The TV and MV flow from 14 normal children was used for comparison. The CMR results were correlated with the findings on echocardiography and electrocardiography.

**Result:**

Delayed onset of the TV flow was observed in 16/31 patients and in none of the controls. The mean delay time was 64.81 ± 27.07 ms (8.7 ± 3.2% of R-R interval). The delay time correlated with the differences in duration of the TV and MV flow (55.94 ± 32.88 ms) (r = 0.90, *p *< 0.001). Delayed onset was associated with prolongation of the RV ejection time in 9 and delayed onset and cessation of the pulmonary arterial flow in 4. Delayed onset was not associated with timing changes in the pulmonary artery in 3. The patients with delayed onset showed reduced RV ejection fraction (p = 0.01). However, the two groups did not show significant differences in TV E/A ratio, ventricular end-diastolic volumes, left ventricular ejection fraction, pulmonary regurgitant fraction, heart rate, PR interval and QRS duration.

**Conclusions:**

Early diastolic dysfunction with delayed onset of TV flow is common after TOF repair, and is associated with reduced RV ejection fraction. It is a further manifestation of interventricular dyssynchrony and represent an additional mechanism of ventricular diastolic dysfunction.

## Background

After repair of tetralogy of Fallot (TOF), dilation and dysfunction of the right ventricle (RV) due to chronic pulmonary regurgitation is very common [1-3]. The manifestations of RV dysfunction include abnormalities of both systole and diastole. Global and regional systolic abnormalities have been detected with almost every imaging modality, and are well documented [1-6]. However, diastolic dysfunction is less well understood, and previous studies have concentrated on markers of decreased late diastolic compliance of the ventricle, as it is reflected by antegrade late diastolic flow in the pulmonary artery [7-10]. Abnormalities of early diastolic function have been less extensively investigated, but shortening of diastolic filling time has been described after repair of TOF [11] and therefore could be an additional mechanism of diastolic dysfunction as has been demonstrated in left-sided lesions such as dilated cardiomyopathy and aortic regurgitation [12,13]. It is also important to understand that abnormalities of the timing and duration of systole adversely affect the timing and efficiency of early diastolic filling. While its mechanism in right-sided lesions has not been clearly explained, abnormalities of systolic and diastolic timing are frequently associated with intra- and interventrcular dyssynchrony. Indeed, there is an increasing awareness of the importance of ventricular dyssynchrony as a mechanism and therapeutic target of ventricular disease [14-16]. In this study we hypothesised that early diastolic function might also be a component of the RV disease associated with late postoperative follow up of TOF. We therefore analysed systolic and diastolic volumes, flows and time intervals obtained during routine phase-contrast imaging at cardiovascular magnetic resonance (CMR), with particular reference to the assessment of tricuspid valve opening as a manifestaion of early diastolic dysfunction.

## Methods

### Patients and control subjects

The study population consisted of 31 patients with repaired TOF who underwent CMR. The patients' demographics and surgical procedures undertaken are summarized in Table 1. The patients were divided into two groups according to the absence (Group I) or presence (Group II) of delayed onset of the TV flow that will be defined later. Controls consisted of 14 children who underwent CMR for a suspected diagnosis or family history of arhythmogenic RV cardiomyopathy but had normal findings. The Research Ethics Board of our institution approved the study.

**Table 1:** Patient characteristics

	Overall	Group I	Group II	*P *value
		
	(n = 31)	(n = 15)	(n = 16)	
Age (years)	12.32 ± 4.10	12.51 ± 3.68	12.15 ± 4.56	NS

Age at operation (years)	1.80 ± 1.66	1.92 ± 2.10	1.70 ± 1.28	NS

Time from operation (years)	9.94 ± 4.30	9.24 ± 4.65	10.59 ± 3.99	NS

No patch	5	2	3	-

RVOT patch	9	3	6	-

Transannular patch	11	6	5	-

Outflow status not known	6	4	2	-

RVOT, right ventricular outflow tract; NS, not significant

### CMR

**Figure 1:**
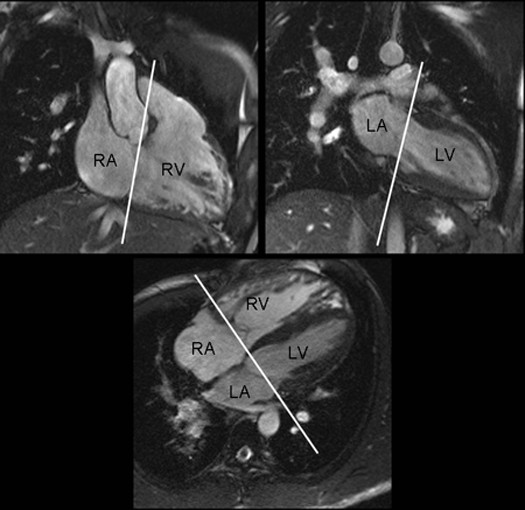
**Reference images for prescription of the atrioventricular valve flow**. End-systolic images of the two-chamber views of the ventricles and four-chamber view are used for prescription of the imaging plane. LA, left atrium; LV, left ventricle; RA, right atrium; RV, right ventricle.

CMR studies were performed on a 1.5 T scanner (Signa CV/*i*; General Electric Medical Systems, Milwaukee, Wisconsin). The imaging sequences included: cine imaging of the ventricles with a balanced steady-state free precession or fast gradient echo sequence in two-chamber, four-chamber and short-axis planes of the ventricles and along the RV outflow tract, cine phase-contrast (PC) velocity mapping of the mid-ascending aorta, main, right and left pulmonary arteries and atrioventricular valves, and contrast-enhanced angiography. Through-plane PC velocity mapping of the atrioventricular valves was prescribed using the end-systolic cine images obtained in four-chamber plane and two-chamber planes of the right and left ventricles (Figure 1). The hinge points of the TV and MV leaflets were used as the reference points for slice prescription. The parameters for PC velocity mapping were adjusted to provide a temporal resolution for > 20 real data points within a cardiac cycle. The parameters for PC velocity mapping were: flip angle of 15° or 20°, repetition time of 6.3 to 14.1 ms, echo time of 3.1 to 5.4 ms, and 2-4 views per segment, providing a temporal resolution of 28 to 112 ms (mean, 48.6 ± 18.6 ms) at a heart rate of 50 to 125 beats per minute. By using a standard interpolation technique, the data were reconstructed into 20-30 phases per cardiac cycle. The velocity encoding limit was set at 150 cm/s and increased if there was aliasing artifact. The slice thickness was 4-5 mm. The matrix and field of view were set to provide an in-plane spatial resolution of 0.9-1.6 × 0.9-2.0 mm.

### CMR Data Analysis

**Figure 2:**
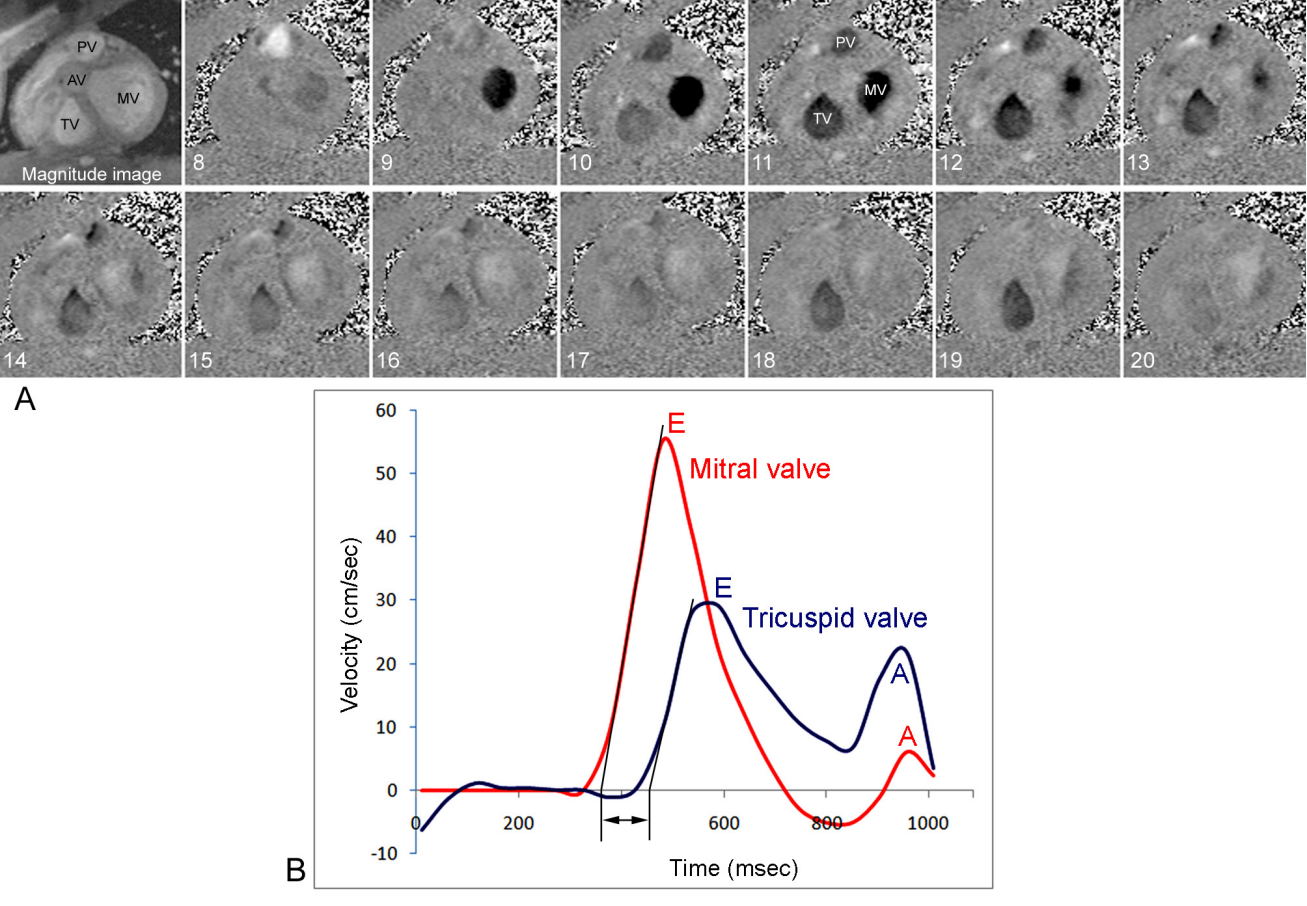
**Phase contrast images (**A**) and time-mean velocity curves (**B**) of the mitral (MV) and tricuspid (TV) valves in diastolic phase**. A magnitude image in left upper corner of **A **shows typical arrangement of the cardiac valves. The remainders are velocity images. The numbers represent the phase numbers in a cardiac cycle. Only the images for phases 8-20 are shown. Black signal in TV and MV areas represent forward flow though the valves. Images 8 and 9 show flow signal in MV area but no signal in TV area. The time-mean velocity curves in **B **show delayed onset and peak of the TV E-wave. The straight elements of the E-waves of the TV and MV flow curves are extrapolated to the baseline and the delay time (double-headed arrow) of the onset of the TV flow is measured. AV, aortic valve; PV, pulmonary valve.

The acquired cine- and PC velocity mapping data were analyzed using a commercially available workstation (Advantage Windows 4.2, GE Medical Systems, Milwaukee, Wisconsin). The inner borders of the TV and MV orifices were manually traced for each image by referencing both magnitude and phase images (Figure 2A), and time-mean velocity curves of the TV and MV flow were generated in a single graph (Figure 2B). The pulmonary arteries and ascending aorta were traced using automatic contour detection algorithm with manual adjustment. Delayed onset of the TV flow was considered significant when the delay was > 20 ms from the onset of the MV flow. From the PC velocity mapping data, the following parameters were derived:

1) Time interval between the onsets of the TV and MV flow (Figure 2B).

2) E- and A-wave peak velocities and E/A ratios of the TV and MV flow.

3) Durations of the diastolic flow through the TV and MV.

4) Durations of the systolic forward flow through the pulmonary artery and ascending aorta.

5) Pulmonary regurgitant fraction.

6) Ejection fractions of the right and left ventricles.

**Figure 3:**
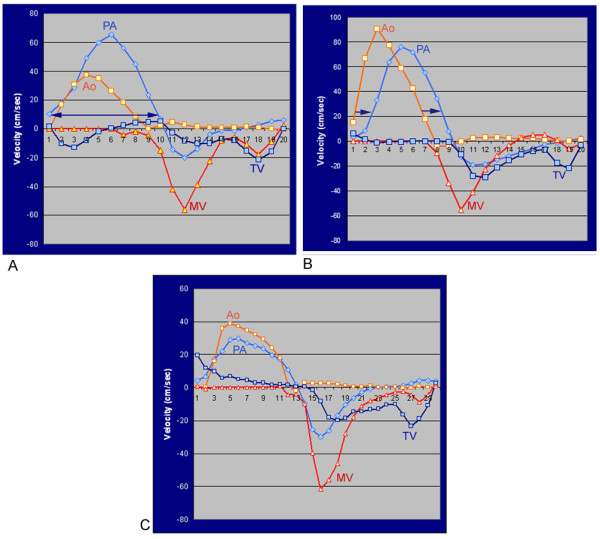
**Three patterns of delayed onset of the tricuspid valve flow**. **A**. *Pattern A*. Delayed onset of the tricuspid valve (TV) flow with prolonged ejection time (double-headed arrow) of the pulmonary arterial (PA) flow as compared to the aortic (Ao)flow. **B**. *Pattern B*. Delayed onset of the TV flow with delayed onset and cessation (arrows) of the PA flow. **C**. *Pattern C*. Delayed onset of the TV flow with synchronous PA and Ao flows. MV, mitral valve.

To assess the timing of the blood flow through the ventricular inlets and outlets, the flows through the TV and MV were compared with the flows through the pulmonary artery and ascending aorta (Figure 3).

Right and left ventricular volumes and ejection fractions were obtained from a short-axis stack of cine images.

### Clinical, Electrocardiographic, Echocardiographic Data Collection

Medical records were reviewed to assess the age at and the type of surgical repair. Electrocardiograms obtained within one year before or after the CMR were reviewed for the assessment of arrhythmias and QRS duration. Electrocardiograms were available in all except one patient. Echocardiograms obtained within one year before or after the CMR were reviewed for the assessment of the presence and severity of tricuspid regurgitation. Echocardiograms were available in all except 5 patients. There were no interventions between the CMR and the ancillary testing in any patient.

### Statistical Analysis

Measured data were expressed as mean ± SD. Unpaired Student t test was performed to compare results between the patients groups. Fisher's exact test was used to assess the group differences for categorical variables. Correlation between different variables was assessed by linear regression analysis and Spearman's correlation analysis. A *p*< 0.05 was considered statistically significant.

## Results

Delayed onset of the TV flow was oberserved in 16 of 31 (52%) patient with repaired TOF and none of the 14 control subjects (*p *< 0.001). There were 15 Group I (without delayed onset of TV flow) and 16 Group II (with delayed onset) patients. There were no significant differences in patients' demographic data between two groups (Table 1).

The mean delay time of the onset of the TV flow in Group II was 64.81 ± 27.07 (28-105) ms, which was 8.7 ± 3.2 (5.0-15.2)% of the R-R interval. The shortest delay time (28 ms) was seen in a patient with a heart rate of 113 beats per minute. The longest time delay (105 ms) was seen in two patients who had the lowest E/A ratio (0.48 and 0.65), the lowest right (31 and 32%) and left (48 and 53%) ventricular ejection fractions, the largest RV end-diastolic volume index (233 and 240 ml/m^2^), and the longest QRS duration (190 and 170 ms).

The delay time of the onset of the TV flow correlated well with the difference in the durations of the TV and MV flow (55.94 ± 32.88 ms) (r = 0.90, *p *< 0.001).

Patients with repaired TOF showed a significalty lower TV E/A ratio than the control subjects (1.02 ± 0.34 versus 1.89 ± 0.67, *p *< 0.001). However, there was no significant difference in TV E/A ratio between two patients groups (Table 2). There was no correlation between the delay time adjusted to the R-R interval and the TV E/A ratio (r = 0.31, *p *= 0.26).

**Table 2:** Comparison of CMR data between Group I and Group II

Parameters		Overall (n = 31)	Group I (n = 15)	Group II (n = 16)	*P *value
Delay of onset of tricuspid valve flow (ms)		-	-	64.81 ± 27.07	-

Tricuspid Valve	E-wave peak velocity (cm/s)	29.47 ± 8.68	32.15 ± 7.02	26.95 ± 9.52	0.10
	
	A-wave peak velocity (cm/s)	31.01 ± 12.76	29.58 ± 8.07	32.26 ± 15.97	0.56
	
	E/A ratio	1.02 ± 0.34	1.12 ± 0.32	0.93 ± 0.34	0.12

Mitral valve	E-wave peak velocity (cm/s)	53.14 ± 8.17	54.02 ± 9.69	52.31 ± 6.67	0.58
	
	A-wave peak velocity (cm/s)	20.00 ± 10.98	17.65 ± 7.12	21.91 ± 13.24	0.31
	
	E/A ratio	3.50 ± 2.00	3.44 ± 1.51	3.54 ± 2.37	0.89

Right ventricle	EDVi (ml/m^2^)	171.66 ± 49.60	174.29 ± 43.82	169.02 ± 56.22	0.77
	
	EF (%)	42.28 ± 8.20	46.06 ± 8.95	38.49 ± 5.35	0.01

Left ventricle	EDVi (ml/m^2^)	83.41 ± 15.32	85.12 ± 16.87	81.69 ± 14.01	0.56
	
	EF (%)	59.72 ± 6.91	58.95 ± 7.25	60.55 ± 6.71	0.54

Pulmonary regurgitant fraction (%)		38.28 ± 10.14	42.23 ± 6.81	35.96 ± 11.41	0.06

Heart rate (beats per minute)		76.80 ± 19.44	77.07 ± 22.40	76.53 ± 16.78	0.94

PR interval (ms)		148.97 ± 32.10	147.21 ± 40.48	150.60 ± 23.11	0.78

QRS duration (ms)		142.33 ± 29.54	136.27 ± 33.17	148.40 ± 25.06	0.27

EDVi, end-diastolic volume index; EF, ejection fraction; MV, mitral valve; TV, tricuspid valve.

Group II showed a significantly lower RV ejection fraction than Group I (*p = *0.01) (Table 2). There was no significant difference in left ventricular ejection fraction or right and left ventricular end-diastolic volumes between two groups.

Signicant pulmonary regurgitation (regurgitant fraction > 10%) was present in 29 patients. Although Group II showed a lower pulmonary regurgitant fraction, the difference did not reach the statistical significance (Table 2). There was no linear relationship between the pulmonary regurgitant fraction and the delay time of the onset of the TV flow (r = 0.37, *p *= 0.157).

All patients were in sinus rhythm. Right bundle branch block was seen in 11 Group I and 14 Group II patients. There was no significant difference in heart rate, PR interval and QRS duration between two groups (Table 2).

Late diastolic antegrade flow in the pulmonary artery was detected in 9 Group I and 8 Group II patients. There was no difference in delay time of the onset of the TV flow between those with and those without late diastolic antegrade flow in the pulmonary artery (71.13 ± 26.67 msec versus 58.25 ± 28.75 msec, *p *= 0.533).

When the atrioventricular valve flows were assessed in conjunction with the flows through the pulmonary artery and ascending aorta, three patterns of delayed onset of the TV flow were recognized (Figure 3):

*Pattern A *(n = 9): delayed onset of the TV flow due to prolonged ejection time with delayed cessation of the forward flow in the pulmonary artery

*Pattern B *(n = 4): delayed onset due to rightward shift of the forward flow curve with delayed onset and delayed cessation of the forward flow in the pulmonary artery

*Pattern C *(n = 3): delayed onset with the forward flows through the pulmonary artery and the ascending aorta occurring synchronously

*Pattern A *was associated with higher incidence (n = 6) of obstruction in the RV outflow tract and/or pulmonary artery than *Patterns B *(n = 1) and *C *(n = 1). *Pattern C *was associated with filling of the RV from the PR before the TV flow starts in all cases. Pulmonary regurgitant fraction was higher in patients with *Pattern *C (mean, 46.7%) than in patients with *Pattern A *or *B *(mean, 35.1% and 26.3%, respectively). *Pattern C *was associated with the largest right ventricular end-diastolic volume index (mean, 197 ml/m^2 ^of body surface area), while *Pattern A *with the smallest volume index (mean, 155 ml/m^2^). *Pattern *C was associated with an intermediate volume (175 ml/m^2^). However, the differences in pulmonary regurgitant fractions and right ventricular end-diastolic volume indices among three patterns were not statistically significant (*p = 0.052 and 0.57, respectively*)

## Discussion

This study demonstrates that delayed onset of the TV flow is seen in approximately half the patients undergoing CMR after repair of TOF. When present, it is associated with decreased RV ejection fraction, suggesting that its mechanism may reflect systolic-diastolic interaction borne out of electrical or mechanical dyssynchrony. Despite its common occurrence, delayed onset of the TV flow has not been previously described, presumably because of the difficulty in obtaining simultaneous atrioventricular flow curves at echocardiography. In our institution we perform through-plane PC velocity mapping across the TV and MV as a routine component of CMR imaging. The imaging plane for PC velocity mapping of the TV and MV can be precisely prescribed using two-chamber views of the right and left ventricles and a four-chamber view obtained at end-systole (Figure 1). Despite slight offset between the planes of the TV and MV, the blood flow through the open valves can be captured simultaneously in all individuals using this maneuver, and therefore is a relatively straightforward new index of ventricular performance. The only exception might be the heart with twisted or criss-cross atrioventricular connection where the opening axes of the atrioventricular valves are not parallel.

In our study, the mechanism of delayed onset of the TV flow varied. Delayed onset of the TV flow was associated with prolonged RV systole (*Pattern A*, Figure 3A) or delayed onsets of both systole and diastole of the RV (*Pattern B*, Figure 3B). It also occurred without delay or prolongation of the systole (*Pattern C*, Figure 3C). *Pattern A *was the commonest. This pattern appears to reflect primarily mechanical events in systole. In two thirds of the patients in this group there was a degree of obstruction of the RV outflow tract and/or pulmonary artery, reflecting the well known association between RV ejection time and afterload [11]. *Pattern B *is most likely reflective of abnormal electro-mechanical coupling. Delay in onset of RV systole, resulting from right bundle branch block or intraventricular conduction delay will likely lead to late cessation of flow and later onset of TV opening. Going along with this, the QRS duration in *Pattern B *was on average 20 ms longer than for *Pattern A*, but this failed to reach statistical significance (P < 0.3). Clearly however, prolongation of QRS under these circumstances is multifactorial, and given the small number of subjects it is unlikely to be exclusively related to timing of onset of systole. Both *Patterns A *and *B *can reasonably be described as manifestations of interventricular dyssynchrony, with abnormalities of systolic timing or duration, imposing secondary effects on early RV diastolic function. This phenomenon is well described for the left ventricle, where post-systolic shortening and left bundle branch block both significantly impose on diastolic function [12,13]. *Pattern C *is not associated with systolic timing events, and may therefore be primarily due to impairment of the early diastolic relaxation of the RV. However, the small number of patients in each group precluded accurate mechanistic analysis.

Pulmonary regurgitant fraction was lower in Group II than in Group I. However, the difference did not reach the statistical significance. It may be speculated that diminished diastolic filling time might allow less time for pulmonary regurgitation. It is a possible explanation for some but unlikely the case of *Pattern C *where pulmonary regurgitation starts before the TV flow starts. *Pattern C *is particularly interesting as the RV is refilled from PR, relatively severe in all cases, before the TV flow starts. This finding implies that the end-systolic pressure is high in the main pulmonary artery. It can also be speculated that delayed onset of the TV flow in *Pattern C *is not significantly affected by other factors such as pulmonary stenosis, right ventricular bundle branch block and impaired myocardial relaxation. Therefore, a high grade pulmonary regurgitation causing excessive dilatation of the RV could be the main factor that delays the onset of the TV flow. However, relation between delayed onset of TV flow, severity of pulmonary regurgitation and RV dilatation should be further assessed on a larger number of patients.

Our data also provide additional insights into the previous studies of abnormalities of systolic and diastolic timing in patients after TOF repair. Isovolumetric relaxation time (IVRT) has been defined echocardiographically as the time interval between the cessation of the RV ejection and the onset of the TV inflow [17-20]. When there is significant pulmonary regurgitation, however, the RV does not stay isovolumetric during the period of echocardiographically defined 'isovolumetric' relaxation time as pulmonary regurgitation starts immediately after the cessation of the forward flow through the pulmonary valve [21]. The data in the previous reports regarding IVRT measured by pulsed Doppler technique in patients with repaired TOF vary widely. Norozi et al [22] reported prolonged IVRT and increased myocardial performance index (MPI) of the RV, while Abd El Rahman et al [18] and Sachdev et al [19] reported a short or negative IVRT and paradoxically decreased MPI in the majority of the patients with repaired TOF. Uebing et al [11] and D'Andrea et al [23] found no significant difference in IVRT between patients with repaired TOF and normal control subjects. Our study showed that the so-called IVRT was short or absent in 13 of 16 patients with delayed onset of the TV flow and short in 8 of 15 patients without delayed onset of the TV flow. Prolongation of this period (*Pattern C*) indicating abnormal relaxation in early diastole was seen in only 3 of 16 patients with delayed onset of the TV flow. It is not clear why the IVRT and MPI vary widely among the published reports, but clearly problems of definition, as they pertain to the technique being used, must be considered. Consequently, the previously published data regarding isovolumetric times and MPI should be assessed and compared cautiously [24-26].

Diastolic dysfunction of the RV has increasingly been recognized as an important factor contributing to long-term morbidity and mortality after repair of TOF [7-10]. Diastolic dysfunction is considered to be due to impaired early relaxation or reduced compliance, or combination of both. Previous studies have concentrated on abnormalities of late diastolic function, as it reflects RV compliance or capacity to further expand, manifest by antegrade diastolic flow in the pulmonary artery [7-10]. Our study showed that delayed onset of the TV flow was associated with significant difference in durations of the TV and MV flow. With the RV diastole ending at the same time with the LV diastole, delayed onset of the TV valve results in shortening of the duration of the TV and, in other words, the RV diastolic filling time. Therefore, delayed onset per se is an additional component of diastolic dysfunction, although delayed onset of the TV flow can be due to systolic RV dysfunction or other mechanisms of diastolic RV dysfunction. As shown in patients with left-sided heart disease [12,13], the effect of diastolic dysfunction due to shortened ventricular filling time can be more pronounced with a high heart rate. Consequently, such abnormalities are most likely functionally correlated with abnormalities of exercise performance, where physiologic increases in heart rate further impose on diastolic filling time. The lack of correlation between delayed onset of tricuspid valve opening and resting functional parameters is perhaps not surprising, and future studies should be designed to assess the relevance of our findings to exercise performance in these patients.

### Study limitations

Our study was a retrospective study and therefore correlation of the CMR parameters with clinical and other imaging data was limited or not possible. In addition, the distribution of the patterns of delayed onset of the tricuspid flow should be assessed among a larger number of patients. Further prospective studies are required to clearly define the mechanism of delayed onset of the TV flow, possibly in conjunction with flow and tissue Doppler echocardiography, and its clinical relevance, particularly in regard to exercise performance as discussed above. Considering that PC velocity mapping is limited by its relatively low temporal resolution, echocardiographic assessment using spectral or tissue Doppler or speckle tracking may provide better differentiation of the right and left ventricular events if the tracing is precisely referenced to simultaneous ECG tracing.

## Conclusions

Delayed onset of the tricuspid valve flow is common in patients with repaired tetralogy of Fallot and is associated with reduced RV ejection fraction. Most frequently it reflects abnormalities of systolic timing and, in turn, abnormal interventricular dyssynchrony resulting from electromechanical coupling. The functional implications of this phenomenon may be limited at rest, but further studies of its importance to exercise performance are warranted..

## List of Abbreviations

CMR: cardiovascular magnetic resonance; IVRT: isovolumetric relaxation time; MPI: myocardial performance index; MV: mitral valve; PC: phase-contrast; PA: pulmonary artery; RV: right ventricle; TOF: tetralogy of Fallot; TV: tricuspid valve

## Competing interests

The authors declare that they have no competing interests.

## Authors' contributions

**AMS: **Study design, Patient identification, analysis and interpretation of data, statistics, drafting of the manuscript. **FA: **nalysis and interpretation of data, statistics, revising the manuscript critically for important intellectual content. **MC: **Analysis and interpretation of data, revising the manuscript critically for important intellectual content. **GB: **ECG data analysis and interpretation, statistics, revising the manuscript critically for important intellectual content. **ANR: **Analysis and interpretation of data, revising the manuscript critically for important intellectual content. **LNB: **Analysis and interpretation of data, revising the manuscript critically for important intellectual content.**CM: **MR data analysis, revising the manuscript critically for important intellectual content. **SJY: **Conception of the study, study design, analysis and interpretation of data, revising the manuscript critically for important intellectual content. All authors read and approved the final manuscript.
